# Gender differences in the impact of retirement on depressive symptoms among middle-aged and older adults: A propensity score matching approach

**DOI:** 10.1371/journal.pone.0212607

**Published:** 2019-03-04

**Authors:** Jin-Won Noh, Young Dae Kwon, Lena Jumin Lee, In-Hwan Oh, Jinseok Kim

**Affiliations:** 1 Department of Healthcare Management, Eulji University, Seongnam, Korea; 2 University Medical Centre Groningen, University of Groningen, Groningen, the Netherlands; 3 Department of Humanities and Social Medicine, College of Medicine and Catholic Institute for Healthcare Management, the Catholic University of Korea, Seoul, Korea; 4 National Institutes of Health Clinical Center, Bethesda, MD, United States of America; 5 Department of Preventive Medicine, Kyung Hee University School of Medicine, Seoul, Korea; 6 Department of Social Welfare, Seoul Women's University, Seoul, Korea; West Chester University of Pennsylvania, UNITED STATES

## Abstract

**Background:**

Retirement is one of the most important later-life status transitions related to changes in workforce participation, lifestyle, and social roles among older adults. The purpose of this study was to investigate the impact of retirement on depressive symptoms by gender in middle-aged and older adult Koreans, using a large, nationally representative sample.

**Methods:**

Using year 2010 and 2012 data from the Korea Longitudinal Study of Aging, we investigated the relationship between adults’ retirement status and depressive symptoms measured by the 10-item Center for Epidemiological Studies–Depression (CES-D10) scale. A series of propensity score matching models were calculated to test the potential retirement effect on adults’ depressive symptoms.

**Results:**

Overall, the level of depressive symptoms of the retired group was not different from that of the employed counterpart. In gender-stratified models, the gain of CES-D10 scores in the retired group was higher than that of the employed group for males, which was in the opposite direction among females. However, the propensity score matching model showed that the relationship between retirement status and CES-D10 score gain was significant for males but not for females.

**Conclusions:**

Our study reinforces the assertion that retirement could worsen depressive symptoms and could differ by gender. Intervention programs focused on the mental health of retired men need to be actively developed and widely implemented.

## Introduction

Retirement is one of the most important later-life status transitions related to changes in workforce participation, lifestyle, and social roles among older adults [1, 2]. Retirement may be associated with poor mental status, including depression, due to reducing the financial stability [3], losing work role and social networks [4], or feeling the gap between the social norms of workforce participation and retirement age [5]. On the other hand, some retired people may feel relief from the pressures of work. They are able to pursue their own interests, and have more autonomy, which could in turn affect their depressive symptoms positively [6], thus, yielding controversial results according to a number of empirical studies. Some researchers suggest that retirement increases the risk of depressive symptoms [3, 7–9], whereas other studies did not observe any association [10], and others reported a decrease in depression after retirement [11–13].

Gender is a key contextual factor influencing the retirement transition because women and men have different workforce attachments [14]. As breadwinners, for instance, men consider work to have a central role in their lives and have a much more continuous employment career. As homemakers, on the other hand, women typically leave the workforce because of marriage or the birth of their children; therefore, they remain out of the labor force, and some even become full-time mothers [15, 16]. Although women’s participation in the labor market has increased recently [17], we would expect that men and women after retirements might experience very different consequences in terms of their mental health problems. For example, men were reported to be substantially stronger than women in the association between retirement and psychological distress [8, 18]. Lee and Smith [8] found a considerable difference in depression rates in men who retired (24% compared to the currently employed 6%); little difference in the level of depression has been shown among women (29% for retired compared to 16% for the currently employed).

Most studies examining the relationships between retirement and depressive symptoms by gender have focused on developed Western countries, and in-depth investigations among Asian populations are limited. Therefore, a population-based study in Korean middle-aged or older people may be helpful in clarifying the association between retirement and depression. Furthermore, most prior studies of retirement have been cross-sectional in nature. However, differences in psychological well-being by retirement status at one point in time may or may not reflect changes in psychological well-being as individuals move from employment to retirement over their life. Dynamic, longitudinal analyses are essential to assess whether snapshot distinctions between retirees and the employed capture any effects of the actual retirement transition. The purpose of this study was to investigate the impact of retirement on depressive symptoms by gender in middle-aged and older adult Koreans, using a large, nationally representative sample.

## Methods

### Data

In this secondary data analysis, we used data from the Korean Longitudinal Study of Ageing (KLoSA). KLoSA asked a series of questions to adults aged from 45 or older at the time of the first interview (2006), regarding multiple facets of their lives, including socio-demographic characteristics, family relationship, health-related issues, and employments. Fully anonymized data from years 2010 and 2012 were combined and used in this analysis. There were 7,920 and 7,486 participants from year 2010 and 2012 periods of KLoSA, respectively, of which 7,134 were included in the final analysis. Only the data from these two waves were used because using data from two different time points from the same respondents was critical for the propensity score matching to be utilized in this analysis. Also, 2010 was the year for the third wave of the KLoSA, which would be expected to produce more reliable observations. This study was approved by the Institutional Review Board of the Seoul Women’s University (IRB-2015A-33) with a waiver for informed consent because the data were obtained from a public data depository.

### Variables

The retirement variable in 2012 was created using questions related to respondents’ employment status in a way that measured whether a respondent experienced her/his retirement between years 2010 and 2012. We coded the retirement variable of a respondent as 1 if she or he answered “not retired” (i.e., currently working or currently not working but looking for jobs) in 2010 and answered “retired” in 2012, and as 0 otherwise.

Respondents’ level of depression was measured using the Korean version of the 10-item Center for Epidemiological Studies–Depression (CES-D10) scale. CES-D10 is composed of 10 questions asking about respondents’ experiences of depressive symptoms during the past week, using a 4-point frequency rating scale [i.e., 0 = rarely (less than one day); 1 = sometimes (from one to two days); 2 = often (from three to four days); 3 = at all times (from five to seven days)]. Respondents’ depression scores were obtained by the total score of the 10 items, which ranged from 0 to 30, with a higher score meaning more severe depressive symptoms. The internal consistency reliability of the CES-D10 scale was measured by Cronbach’s alpha, which ranged from 0.861 in 2010 to 0.853 in 2012.

Respondents’ age was calculated using their report of birth year. Respondents’ education level was self-reported and categorized as elementary level or lower, middle school graduate, high school graduate, and college graduate or higher. Gender (0 = male; 1 = female), marital status (0 = currently not married; 1 = currently married), self-rated health status using a 5-point scale (1 = very bad; 5 = very good), and living area (0 = not urban; 1 = urban) were self-reported.

### Statistical analysis

Propensity score matching (PSM) has been widely used to estimate causal treatment effects when experimental designs with randomization were not feasible [19]. It is not deemed realistic to assign study participants randomly to either a retirement group or a nonretirement group in our study; hence, we used a PSM approach to examine the influence of retirement on middle-aged or older adults’ level of depressive symptoms. PSM allows us to contrast the level of depressive symptoms of those who experienced retirement and did not experience it but who were considered to have the same or nearly the same probability from retirement (propensity) based on a wider range of relevant characteristics such as age, gender, education level, marital status, living area, health status, and level of depression. We calculated the average treatment effect on the treated (retirement) group to test whether retirement status was associated with a respondent’s level of depression. Stata 13.2 (StataCorp LP, College Station, Texas) was used to manage and analyze the data for this study.

## Results

### Comparison between retired and employed groups

Table 1 summarizes the comparison of retired and employed groups in terms of their levels of depressive symptoms measured by CES-D10 scores. Results showed that overall, the CES-D10 scores of the retired group were not different from those of the employed group in both 2010 (t = 0.47, p = 0.640) and 2012 (t = 1.24, p = 0.213) or, in terms of the gain of CES-D10 scores, the difference of 2012 scores from 2010 scores (t = 0.68, p = 0.494).

**Table 1:** CES-D10 score change by retirement status between 2010 and 2012.

	Retired between 2010 and 2012			Not retired between 2010 and 2012			
	Mean	SD	95% CI	Mean	SD	95% CI	*t*
Total							
CES-D10 score gain	0.17	5.18	(–0.26, 0.59)	0.01	5.08	(–0.11, 0.14)	0.68
CES-D10 score 2010	7.41	5.51	(6.96, 7.87)	7.53	5.64	(7.40, 7.66)	0.47
CES-D10 score 2012	7.61	5.56	(7.15, 8.07)	7.31	5.38	(7.18, 7.45)	1.24
Male							
CES-D10 score gain	1.14	5.11	(0.56, 1.72)	0.01	4.77	(–0.17, 0.19)	3.86***
CES-D10 score 2010	6.45	4.85	(5.90, 7.00)	6.92	5.39	(6.73, 7.12)	1.46
CES-D10 score 2012	7.60	5.55	(6.97, 8.23)	6.66	5.03	(6.47, 6.85)	3.04**
Female							
CES-D10 score gain	–0.93	5.05	(–1.54, –0.32)	0.02	5.29	(–0.15, 0.19)	2.82**
CES-D10 score 2010	8.49	5.99	(7.77, 9.21)	7.97	5.78	(7.80, 8.15)	1.42
CES-D10 score 2012	7.62	5.59	(6.94, 8.29)	7.79	5.57	(7.61, 7.97)	0.50

**Notes:** CES-D10, 10-item Center for Epidemiological Studies–Depression; SD, standard deviation; CI, confidence interval

*p <0.05

**p <0.01

***p <0.001

However, results from a gender-stratified analysis of the same relationship revealed that, for males, the gain of CES-D10 scores from the retired group [mean (SD) = 1.14 (5.11), 95% CI = (0.56, 1.72)] was indeed higher than the same of the employed group (t = 3.86, p < 0.001). For females, we found a significant difference between the retired and employed groups (t = 2.82, p = 0.005) but in the opposite direction. Specifically, among the females who retired between 2010 and 2012, the CES-D10 scores decreased in the same period [mean (SD) = –0.93 (5.05), 95% CI = (–1.54, -0.32)] (Table 1).

### PSM model

We used a set of relevant covariates, including age, gender, marital status, education level, self-rated health status, urban/rural status of the residential area, and CES-D10 score in 2010 to predict propensity scores of retirement between 2010 and 2012. Table 2 shows the balance between retirement and non-retirement groups in terms of the covariates considered in this analysis that are shown before and after the propensity score matching, respectively. The results showed that, as a result of PSM, the discrepancy between retirement and non-retirement groups in terms of age (t = 2.40, p = 0.016), gender (Chi-squared (1) = 23.9, p < 0.001), and marital status (Chi-squared (1) = 4.61, p = 0.032) became insignificant after the matching, which indicated the PSM being effective.

**Table 2:** Covariate balance before and after matching.

Variable	Category	Before matching					After matching				
		M	SD		*t*	*p*	M	SD		*t*	*p*
Age	Not retired	66.2	10.7		2.40	0.016	66.8	9.5		0.94	0.349
	Retired	67.3	9.1				67.3	9.1			
Self rated health	Not retired	3.0	0.9		0.05	0.962	3.0	0.9		1.02	0.307
	Retired	3.0	0.9				3.0	0.9			
CES-D10 (2010)	Not retired	7.5	5.6		0.59	0.557	7.2	5.4		0.55	0.584
	Retired	7.4	5.5				7.4	5.5			
		%			Chi-squared	*p*	%			Chi-squared	*p*
		Female									
Gender^1^	Not retired	57.7			23.9	<0.001	46.8			0.02	0.878
	Retired	47.2					47.2				
		Married									
Marital status^2^	Not retired	76.4			4.61	0.032	83.3			1.66	0.198
	Retired	80.3					80.4				
		Urban									
Urban^3^	Not retired	74.6			1.08	0.299	75.7			0.10	0.757
	Retired	76.6					76.6				
		1	2	3			1	2	3		
Education^4^	Not retired	47.0	16.8	26.8	5.06	0.167	46.2	18	25.4	1.30	0.730
	Retired	47.6	16.2	24.3			47.6	16.2	24.3		

1, 2, 3, 4: Reference categories of gender, marital status, urban status, and education were male, not married, not urban, and college degree or higher, respectively(% not shown)

4: For education 1 =“Elementary school graduate or lower”, 2 =“Middle school graduate”, 3 =“High school graduate”.

CES-D10, 10-item Center for Epidemiological Studies–Depression; M, mean; SD, standard deviation; CI, confidence interval

Table 3 shows the relationship between retirement status and level of depressive symptoms based on different analytic approaches, including regression without covariates (OLS), regression with a set of relevant covariates (OLS adjusted), and propensity score matching (PSM). Results showed that, with PSM models involved, there was no significant difference between retired and employed groups in terms of their CES-D10 scores’ gain in average treatment effect among the retired group (ATET) (B (SE) = 0.44 (0.29), p = 0.125). However, the results showed that this retirement effect on CES-D10 score gain was different by gender. Specifically, the relationship between retirement status and CES-D10 score gain was significant for males (B (SE) = 0.75 (0.36), p = 0.037) but not for females (B (SE) = –0.57 (0.38), p = 0.135) (Table 3).

**Table 3:** Estimated effects of retirement status change between 2010 and 2012 on CES-D10 score.

	OLS (N = 7,134)			OLS (adjusted)^a^ (N = 7,134)			PS-matched ATET^b^		
	B	SE (B)	95% CI	B	SE (B)	95% CI	B	SE (B)	95% CI
CES-D10 score gain									
All	0.15	0.22	(–0. 29, 0.58)	0.12	0.19	(–0.26, 0.50)	0.44	0.29	(–0.12, 1.00)
Male	1.13***	0.29	(0.56, 1.71)	0.90***	0.26	(0.40, 1.41)	0.75*	0.36	(0.05, 1.46)
Female	-0.92**	0.33	(-1.57, -0.26)	-0.56	0.29	(-1.12, 0.01)	–0.57	0.38	(–1.32, 0.18)
CES-D10 score 2012									
All	0.26	0.24	(–0.21, 0.72)	0.12	0.19	(–0.26, 0.50)	0.68*	0.28	(0.13, 1.23)
Male	0.94**	0.31	(0.34, 1.55)	0.90***	0.26	(0.40, 1.41)	1.01**	0.41	(0.20, 1.82)
Female	-0.19	0.35	(-0.88, 0.51)	-0.56	0.29	(-1.12, 0.01)	–0.39	0.41	(–1.20, 0.42)

Notes

a: adjusted for age, gender, education, marital status, self-rated health status, urbanity, and CES-D10 score in 2010

b: treatment-effects estimation for observational data, using propensity score matching method

CES-D10, 10-item Center for Epidemiological Studies–Depression; OLS, ordinary least squares; PS, propensity score; ATET, average treatment effects for the treated, i.e., retired; SE, standard error; CI, confidence interval

*p <0.05

**p <0.01

***p <0.001

## Discussion

In this study, the impact of retirement on depressive symptoms was analyzed using a CES-D10 score. In results, the CES-D10 score did not show a significant difference between retired and employed groups, but the result differed by gender. The CES-D10 score increase during the retirement process was larger in the retired male group than in the employed male group. On the contrary, CES-D10 score did not show any difference in the female group. Further, regression and PSM were conducted to minimize the selection bias between the retired and employed groups. OLS and PSM showed similar results. The CES-D10 score was higher in the retired male group after retirement. Retirement increased CES-D10 scores after adjustment in the male group. Conversely, the effect of retirement was not shown in the female group even after adjustment.

Previous studies on the effects of retirement on health have reported mixed results [20]. Some studies showed improved health results after retirements. For example, one study found that the subjective health status improved after retirement [21], and that retirement was associated with 11–13% reduction in headache prevalence [22]. On the contrary, other studies highlighted the negative impact of retirement on health. For example, the risk of stroke and myocardial infarction was increased after retirement [23]. The effect of retirement on mental health was also reported as mixed. Statutory retirement and early voluntary retirement are related not only to better physical functioning but also better mental health [24], and the use of antidepressants decreased after retirement in a Finnish study [11]. However, other studies found no significant relationship, or even a negative relationship between retirement and mental health [25, 26]. For example, the prevalence of hospital treatment due to depression increased after retirement. Retirement also did not reduce the prevalence of purchase of antidepressants [11]. Other studies found similar results; age-related retirement was not related to decreased use of antidepressants [25]. For these variations in the effects of retirement on health, some explanation is added. For example, one study emphasized the importance of the timetable. In other words, early retirement could be bad for health, but on-time retirement was not related to health disadvantages [27].

Gender differences in the impact of retirement on various health outcomes are noteworthy. In this study, the increase in the CES-D10 score after retirement was shown only in males. Females’ CES-D10 scores did not change after retirement. The difference by gender was also found in other studies [28, 29]. For example, retirement was associated with physical dysfunction in both males and females in an Australian study, but the effect of retirement on psychological distress was limited only to males. Females were not affected by retirement in terms of psychological distress [30]. The reason for retirement was also found to be important in a study; involuntary retirees feel worse health after retirement than voluntary retirees [31].

Studies on the relationship between retirement and health outcomes have been conducted in Korea. One study asserted that involuntary job loss and voluntary retirement increased the risk of cerebrovascular disease in males, although the effect of involuntary job loss was greater than that of voluntary retirement [28]. In females, however, involuntary job loss did not increase the risk of cerebrovascular disease. Also, the associations between job loss and health risks were weaker in females than in males [28]. Another study found that the effect of retirement on depressive symptoms was greater in cases of involuntary retirement than in voluntary ones. In addition, females were negatively affected by the spouse’s voluntary retirement. On the contrary, the males were not affected [29]. These results showed that the effect of retirement differed by gender and by the specific circumstances of retirement in Korea

Health itself may influence decisions about retirement [21], so health status between retiree groups and employed groups could be different. These could affect interpretation of the causes of retirement. In this study, use of longitudinal data minimized the potential impact of reverse causation, that is, that mental health conditions led to retirement. To adjust the effects of other confounding variables, OLS and PSM were conducted and compared with each other. Both models showed similar results. In males, the retired group exhibited high CES-D10 scores compared with the employed group, and retirement was found to be associated with increased CES-D10 score. However, in females, the CES-D10 score did not differ after retirement. Our result reaffirms previous studies conducted in Korea and other countries which found that depressive symptoms in retired males are more severe than in retired females [29, 30]. These gender differences assert that the preretirement environment is different between genders. Females focused more on home compared to males; therefore, retirement may be less stressful for females than for males [31]. In other words, males may perceive job loss as social failure, but females could move their attention from work to home [28]. This explanation on gender differences may be reinforced by the tradition of patriarchal Confucianism in Korea [29]. In the tradition of patriarchal Confucianism, males concentrate on the job, and retirement takes away the motivation for productive activity in males much more than it does in females. Although the tradition of Confucianism has weakened, our study was limited to 45-year-old and older groups, and the traditional gender role still can be powerful in this age group [29]. In contrast, the results of this study are different from other studies showing that retirement improves mental health [11, 24]. These could result from the fact that pensions and other social security systems in Korea are deficient compared to other Organisation for Economic Co-operation and Development (OECD) member countries [28].

Our study has some limitations. First, we could not classify voluntary and involuntary retirement due to the limitation of data. Another limitation is that our study period was a relatively short time. Because the positive effect of retirement on health is supposed to last only a short time, even if positive effects exist, the health status of those in the retired group could be worse. Indeed, gender differences might be driven by differences in labor market attachment and selection into certain occupations could be considered in the future study. In this study, by using nationally representative data and PSM, we found that retirement is associated with depressive symptoms, which is different by gender. Retirement is related with an increase of the depression score in males. In females, retirement is not related with an increase of the depression score. Our study reinforces the assertion that retirement could worsen depressive symptoms and could differ by gender. Therefore, male retirees’ well-being could decrease and their medical expenditures are likely to increase. Intervention programs focused on the mental health of retired men need to be actively developed and widely implemented.

## References

[1] Ekerdt DJ. Frontiers of research on work and retirement. J Gerontol B Psychol Sci Soc Sci. 2010; 65B: 69–80. 10.1093/geronb/gbp109 20008480

[2] Szinovacz ME. Female retirement: Effects on spousal roles and marital adjustment. J Fam Issues. 1980; 1: 423–40. 10.1177/0192513X8000100307

[3] Kim JE, Moen P. Retirement transitions, gender, and psychological well-being: A life-course, ecological model. J Gerontol B Psychol Sci Soc Sci. 2002; 57: 212–22. 11983732 10.1093/geronb/57.3.p212

[4] van Solinge H, Henkens K. Adjustment to and satisfaction with retirement: Two of a kind? Psychol Aging. 2008; 23: 422–34. 10.1037/0882-7974.23.2.422 18573015

[5] Clarke P, Marshall V, House J, Lantz P. The Social Structuring of Mental Health over the Adult Life Course: Advancing Theory in the Sociology of Aging. Soc Forces. 2011; 29: 1287–313. 10.1353/sof.2011.0036 22081728 PMC3210581

[6] Drentea P. Retirement and mental health. J Aging Health. 2002; 14: 167–94. 10.1177/089826430201400201 11995739

[7] Hamilton VH, Merrigan P, Dufresne E. Down and out: estimating the relationship between mental health and unemployment. Health Econ. 1997; 6: 397–406. 9285232 10.1002/(sici)1099-1050(199707)6:4<397::aid-hec283>3.0.co;2-m

[8] Lee J, Smith JP. Work, retirement, and depression. J Popul Ageing. 2009; 2: 57–71. 10.1007/s12062-010-9018-0 23687521 PMC3655414

[9] Szinovacz ME, Davey A. Retirement transitions and spouse disability: effects on depressive symptoms. J Gerontol B Psychol Sci Soc Sci. 2004; 59: S333–S42. 15576864 10.1093/geronb/59.6.s333

[10] Latif E. The impact of retirement on mental health in Canada. J Ment Health Policy Econ. 2013; 16: 335–46. 23676414

[11] Oksanen T, Vahtera J, Westerlund H, Pentti J, Sjösten N, Virtanen M, et al Is retirement beneficial for mental health?: Antidepressant use before and after retirement. Epidemiology. 2011; 22: 553–9. 10.1097/EDE.0b013e31821c41bd 21502864 PMC3132597

[12] Vahtera J, Westerlund H, Hall M, Sjösten N, Kivimäki M, SalO P, et al Effect of retirement on sleep disturbances: the GAZEL prospective cohort study. Sleep. 2009; 32: 1459–66. 19928385 10.1093/sleep/32.11.1459 PMC2768952

[13] Westerlund H, Vahtera J, Ferrie JE, Singh-Manoux A, Pentti J, Melchior M, et al Effect of retirement on major chronic conditions and fatigue: French GAZEL occupational cohort study. BMJ. 2010; 341: c6149 10.1136/bmj.c6149 21098617 PMC2990862

[14] Schulz JH, Binstock RH. Aging Nation: The Economics and Politics of Growing Older in America. Westport, CT: Greenwood Publishing Group; 2006.

[15] Moen P. Women’s two roles: A contemporary dilemma. New York: Auburn House; 1992.

[16] Quick HE, Moen P. Gender, employment, and retirement quality: a life course approach to the differential experiences of men and women. J Occup Health Psychol. 1998; 3: 44–64. 10.1037/1076-8998.3.1.44 9552271

[17] International Labour Organization. Global employment trends 2014: The risk of a jobless recovery. Geneva: International Labour Organization; 2014 http://www.ilo.org/global/research/global-reports/global-employment-trends/2014/WCMS_233953/lang—ja/index.htm

[18] Vo K, Forder PM, Tavener M, Rodgers B, Banks E, Bauman A, et al Retirement, age, gender and mental health: findings from the 45 and up study. Aging Ment Health. 2015; 19: 647–57. 10.1080/13607863.2014.962002 25271125

[19] Guo S, Fraser M. Propensity score analysis: Statistical methods and applications. 2nd Ed Los Angeles, CA: Sage; 2014.

[20] van der Heide I, van Rijn RM, Robroek SJ, Burdorf A, Proper KI. Is retirement good for your health? A systematic review of longitudinal studies. BMC public health. 2013; 13: 1180 10.1186/1471-2458-13-1180 24330730 PMC4029767

[21] Eibich P. Understanding the effect of retirement on health: Mechanisms and heterogeneity. J Health Econ. 2015; 43: 1–12. 10.1016/j.jhealeco.2015.05.001 26079117

[22] Sjösten N, Nabi H, Westerlund H, Singh-Manoux A, Dartigues JF, Goldberg M, et al Influence of retirement and work stress on headache prevalence: a longitudinal modelling study from the GAZEL Cohort Study. Cephalalgia. 2011; 31: 696–705. 10.1177/0333102410394677 21220374 PMC3317892

[23] Moon JR, Glymour MM, Subramanian SV, Avendaño M, Kawachi I. Transition to retirement and risk of cardiovascular disease: Prospective analysis of the US health and retirement study. Soc Sci Med. 2012; 75: 526–30. 10.1016/j.socscimed.2012.04.004 22607954 PMC3367095

[24] Jokela M, Ferrie JE, Gimeno D, Chandola T, Shipley MJ, Head J, et al From midlife to early old age: health trajectories associated with retirement. Epidemiology. 2010; 21: 284–90. 10.1097/EDE.0b013e3181d61f53 20220519 PMC3204317

[25] Olesen K, Rod NH, Madsen IE, Bonde JP, Rugulies R. Does retirement reduce the risk of mental disorders? A national registry-linkage study of treatment for mental disorders before and after retirement of 245,082 Danish residents. Occup Environ Med. 2015; 72: 366–72. 10.1136/oemed-2014-102228 25814269 PMC4413684

[26] Leinonen T, Lahelma E, Martikainen P. Trajectories of antidepressant medication before and after retirement: the contribution of socio-demographic factors. Eur J Epidemiol. 2013; 28: 417–26. 10.1007/s10654-013-9792-0 23508328

[27] Calvo E, Sarkisian N, Tamborini CR: Causal effects of retirement timing on subjective physical and emotional health. J Gerontol B Psychol Sci Soc Sci. 2013; 68: 73–84. 10.1093/geronb/gbs097 23149431

[28] Kang MY, Kim HR: Association between voluntary/involuntary job loss and the development of stroke or cardiovascular disease: a prospective study of middle-aged to older workers in a rapidly developing Asian country. PLoS One. 2014; 9: e113495 10.1371/journal.pone.0113495 25409032 PMC4237425

[29] Park H, Kang MY. Effects of voluntary/involuntary retirement on their own and spouses' depressive symptoms. Compr Psychiatry. 2016; 66: 1–8. 10.1016/j.comppsych.2015.11.009 26995229

[30] Byles JE, Vo K, Forder PM, Thomas L, Banks E, Rodgers B, et al Gender, mental health, physical health and retirement: A prospective study of 21,608 Australians aged 55–69 years. Maturitas. 2016; 87: 40–8. 10.1016/j.maturitas.2016.02.011 27013287

[31] van Solinge H. Health change in retirement: a longitudinal study among older workers in the Netherlands. Res Aging. 2007; 29: 225–56. 10.1177/0164027506298223

